# Radiological determination of the cranial index of present-day Ghanaians

**DOI:** 10.1080/20961790.2021.1886680

**Published:** 2021-03-31

**Authors:** Benard Ohene Botwe, Jeffrey Nana Afari Boadu, Kofi Adesi Kyei, John D., Thompson

**Affiliations:** a Department of Radiography, University of Ghana School of Biomedical & Allied Health Sciences, College of Health Sciences, Accra, Ghana; b Directorate of Radiography, School of Health Sciences, University of Salford, Salford, UK

**Keywords:** Forensic sciences, cranial index, CI, sexual dimorphism, computed tomography, CT

## Abstract

The cranial index (CI) of Ghanaians is currently unknown. The aim of this study was to measure the CI in a population of Ghanaians in order to classify them against pre-determined CI categories. A systematic random sampling method was used to select 300 normal computed tomography (CT) head scans of adult Ghanaians from the largest hospital in Ghana. All patients were deemed to have a normal cranial image configuration based on the radiological report. The biparietal diameter (BPD, width) and the occipitofrontal diameter (OFD, length) were measured on transaxial CT images using a workstation with a calibrated measurement calliper tool. The CI ratio was calculated as the BPD multiplied by 100 and divided by the OFD. Mean, standard deviation (SD) and range were calculated for BPD, OFD and CI. Differences in measurements between demographic groups were compared using an unpaired *t*-test, with test *α* set at 0.05. Of the population of Ghanaians included in this study, 165 (55%) were male and 135 (45%) were female. The mean CI was 77.3 ± 3.6 in males and 79.0 ± 3.3 in females, placing both genders in the mesocephalic category. However, the difference between males and females was found to be statistically significant (*P* = 0.02). The study indicated that most Ghanaian adults belong to the mesocephalic category of CI. Females also had a higher CI, which could be used to differentiate gender groups. This information can be useful for forensic medicine, plastic surgeries for clinical and research purpose.

**Key points**
- This study found the mean CI of adult Ghanaians to be 78.0 ± 13.0.
- This indicates that most Ghanaian adults belong to the mesocephalic category of CI.
- Females had a higher CI, which could be used to differentiate gender groups.
- To the best of our knowledge, this is the first study which assessed CI of Ghanaians using CT scan.

## Introduction

The cranial index (CI) (also known as the cephalic index) is a useful anthropological measure to help determine racial and sexual differences [1, 2]. The biometric features of the cranium can be used in the assessment of racial backgrounds, growth and development, and the diagnosis of any abnormalities in cranial size and shape within a population [3, 4]. In addition, CI can be particularly valuable in the identification of unknown remains in crime scene [5]. There are six classifications of CI which are summarised in Table 1.

**Table 1.** The six classifications of the cranial index (CI) and the shape of the skull when viewed from the top [6], cited with permission.

Classification	CI	Shape
Hyperdolichocephalic	65.5–69.9	Long and narrow when seen from the top
Dolichocephalic	70.0–74.9	
Mesocephalic	75.0–79.9	Nearly oval when seen from the top
Brachycephalic	80.0–84.9	Broad and short skull when seen from the top
Hyperbrachycephalic	85.0–89.9	
Ultrabrachycephalic	90.0+	

Forensic anthropology is a difficult task, particu­larly when assessing human remains in an advanced stage of decomposition. In complex situations, such as the evaluation of human remains in a mass grave, it can be particularly useful to have metrics that can be used to identify the victims as a particular race and gender, especially in countries like Ghana, where there is no national DNA database to rely on. The CI has also been found to help identify remains and provide cranial morphometry between parents and offspring which would help in the identification of genetic character transmission [1].

CI studies in countries like Japan, China, Nigeria, India and the USA among others, have classified their populations based on their average cranial indices [3, 7]. These developed indices have given them the advantage of using the CI as a reference point in forensic study and the study of sexual differences among individuals from different backgrounds. However, the CI has not been established for Ghanaian natives and it would be useful to help in planning of complex surgeries for craniofacial injuries [8]. It could also be used in assessing the growth and development of individuals and in the diagnosis of abnormalities of cranial shape and size [4]. Though CI is an important marker in the evaluation of racial, gender differences and a supporting tool in medicine, little is known about the CI and its variations in Ghanaians.

This study uses a modern technology, computed tomography (CT), to measure the CI in a population of Ghanaians. Varying approaches have been used to establish CI, including measurement on dry bones, projectional X-ray imaging, and cross-sectional imaging [5] but the geometrical accuracy of transaxial CT images was preferred for this study[9, 10].

## Methods and materials

In accordance with established research protocols, ethical approval was granted by the Ethics and Protocols Review Committee of the School of Biomedical and Allied Health Sciences of the University of Ghana for the commencement of this study. Data collection was also approved by Korle Bu Teaching Hospital, where all images were acquired between 1st August 2018 and 30th April 2019 with patients’ written informed consents. This is the largest hospital in Ghana, receiving referrals from all parts of the country.

### Patients

Sample size was determined using a calculation recom­mended by Charan and Biswas [11] to help generalise the results to the overall population of Ghana. Data from a total of 300 patients were collated using a systematic random sampling method. Only patients of 18 years and older were included in the study. Adult skull images were used because it was assumed that the skull vault has fused [12]. All patients were deemed to have a normal cranial image configuration based on the radiological report. Patients of foreign origin were excluded from the study. Demographic data, including sex and age of the sample were recorded. Names and personal identifiers were not recorded so that the data remained anonymous. No images were retained following measurement.

### Image acquisition & measurement

Images were acquired using a Toshiba Aquilion ONE CT scanner (Toshiba Medical Systems Corporation,Otawara, Japan) using a standard acquisition protocol. Details of the protocol used for the head CT procedures are presented in Table 2.

**Table 2.** The standard acquisition protocol used to perform the head computed tomography (CT) scans.

Acquisition parameter	Value
Tube current (kVp)	120
Tube current modulation	Sure exposure 3 D
Pitch	0.656
Rotation time (s)	0.75
Detector configuration	32 × 0.5
Scan range	Base of skull to vertex
Slice thickness (mm)	0.5

Measurements of the skull were performed on workstation software (Version 4.82, ER001) on transaxial image of the skull. The biparietal diameter (BPD, width), defined as the distance between most projecting points at either side of the skull, which is usually just superior and posterior to the external auditory meatus (EAM), and the occipitofrontal diameter (OFD, length), defined as the distance from the glabella to the most projecting point at the back of the skull. Both measurements were made using a digital calliper within the workstation software to calculate the CI, which is expressed as: CI =BPD/OFD × 100.

### Data analysis

Data were recorded for all patients and descriptive statistics produced in Microsoft Excel©. Mean, standard deviation (SD) and range were calculated for BPD, OFD and CI. Differences in measurements between demographic groups (male and female) were compared using a Mann-Whitney U test with differences considered to be statistically different at a test *α* of 0.05. A Shapiro-Wilk test was used to assess normality of the data.

## Results

### Demographics and normality of data

**Figure 1.**
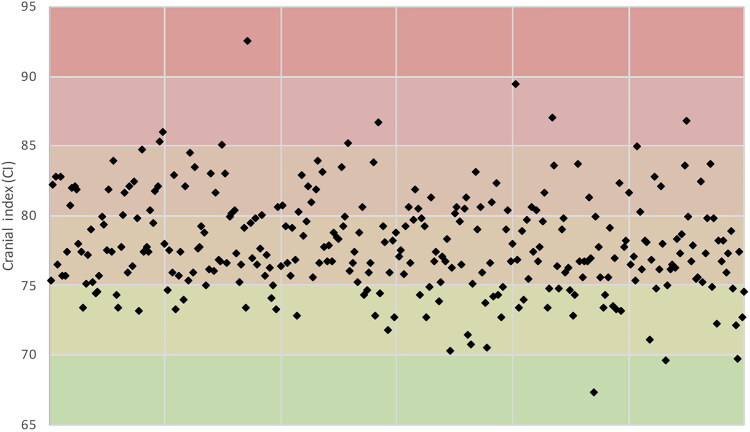
The distribution of all measurements of cranial index for the 300 subjects included in this study. As can be seen from this graphic, the majority of Ghanaians fall into the mesocephalic category (75.0–79.9).

Data from 300 CT head images were used to gene­rate the mean CI of Ghanaian nationals. Within our sample there were 165 males (55%) and 135 females (45%). The age range was 18–94 years with mean of (49.0 ± 18.1) years old. A Kolmogorov-Smirnov test revealed that the age range of the sample was not normally distributed (*P* = 0.042, skewness = 0.11, kurtosis = −0.92), therefore a Mann-Whitney U Test was used for subsequent tests comparing males and females as it does make assumptions about normality. The distribution of all subjects included can be found in Figure 1.

### BPD, OFD and CI

The mean BPD, OFD and CI values observed for the entire sample were (139.4 ± 5.4) mm (range of 126.5–157.2 mm), (179.1 ± 8.6) mm (range 157.6–201.3 mm) and 78.0 ± 13.0 (range 67.4–92.6), respectively. In relation to gender, the mean BPD, OFD and CI values obtained for males were (140.5 ± 5.6) mm (range 126.5–157.2 mm), (182.0 ± 8.5) mm (range 157.6–201.3 mm) and 77.3 ± 3.6 (range 67.4–89.5), respectively. The female group also recorded mean BPD, OFD and CI values of (138.0 ± 4.9) mm (range 129.1–152.2 mm), (175.6 ± 7.4) mm (range 157.6–201.3 mm) and 79.0 ± 3.3 (range 72.9–92.6), respectively.

**Table 3.** Comparison of biparietal biameter (BPD), occipitofrontal biamete (OFD) and cranial index (CI) for male and female Ghanaians.

Parameter	Sex	Mean ± SD	95% Confidence interval	Mean difference (95% confidence interval)	*P*-value
OFD (mm)	Male	182.0 ± 8.5	139.3–141.2	6.1 (4.2–8.1)	<0.001
	Female	175.6 ± 7.4	174.3–176.8		
BPD (mm)	Male	140.5 ± 5.6	139.3–141.2	2.32 (1.0–3.5)	<0.001
	Female	138.0 ± 4.9	137.2–138.8		
CI	Male	77.3 ± 3.6	76.7–77.9	1.4 (0.5–2.3)	<0.001
	Female	79.0 ± 3.3	78.2–79.3		

**Table 4.** The frequency of ages in the sample and the mean cranial index (CI) for males and females in each age category.

Sex	Age (years)	Frequency	CI (mean ± SD)	Range	P-value
Male	18-25	22	79.1 ± 3.3	72.8–89.5	0.106
	26-35	30	78.1 ± 2.8	72.7–87.1	
	36-45	28	77.3 ± 2.9	67.4–86.7	
	46-55	27	76.7 ± 2.9	69.7–83.7	
	56-65	19	72.2 ± 3.1	69.8–83.9	
	66-75	25	73.3 ± 2.8	70.6–81.0	
	76+	14	77.3 ± 3.7	74.0–80.0	
Female	18-25	12	79.1 ± 3.9	75.3–84.6	0.114
	26-35	20	79.2 ± 3.4	73.2–84.8	
	36-45	18	78.5 ± 3.5	72.9–85.4	
	46-55	33	79.4 ± 2.7	74.1–92.6	
	56-65	25	79.0 ± 3.1	74.5–86.0	
	66-75	18	76.6 ± 3.4	73.4–81.8	
	76+	9	78.4 ± 4.0	73.3–85.1	

In this study, the majority of measurements (*n* = 163, 54.3%) were classified as mesocephalic. Others were: brachycephalic (*n* = 71, 23.7%), dolichocephalic (*n* = 53, 17.7%), hyperbrachycephalic (*n* = 9, 3.0%), hyperdolichocephalic (*n* = 3, 1.0%) and ultrabrachycephalic (*n* = 1, 0.3%).

## Discussion

The cranium is the part of the skeleton that forms the cavity of the brain. A good knowledge of its morphology presents an important character in the determination of racial and gender differences [2, 13]. Apart from the CI being importance in forensic studies, it also crucial for the assessment of pre- and post-operative correction of skull deformations [9]. This study was aimed at determining the CI of Ghanaians using CT scan images. The CT images are considered a very accurate and good alternative in the absence of skeletal remains [14]. From the study, the mean CI was 78.0 ± 13.0 to suggest that most of the participants were mesocephalic (CI of 75.0–79.9), with their skulls nearly oval [6].

The mean CI in males was ± 77.3 ± 3.6 while that of females was 79.0 ± 3.3 both being in the mesocephalic group. According to a study [15], differences are generally expected between males and females, with CI found to be larger in female. This result supports prior findings of other studies such as that of Munguti et al. [16], which produced CI of 72.34 ± 4.34 in females and 71.04 ± 3.58 in males. However, the findings in the present study contrast with of that of Oladipo and Olotu [17], in which males of the Ijaw ethnic group in Nigeria were hyperbrachycephalic while females were mesocephalic. Oladipo et al.’s [13] findings on Ibibio tribe also indicated that Ibibio males had higher CI than females.

This study established the mean values of the CI for various age groups in both genders. The results showed the difference between the CI of the various age groups, with respect to gender. The difference was observed to be statistically insignificant in both males and females, respectively. This indicates that the various age groups all have almost the same CI. Moreover, there was no particular pattern between the age groups and their cranial indices. This result is in agreement with findings by Oladipo et al. [13] and Osunwoke et al. [18] on Ogonis and Ogu and Ikwerre ethnic groups, identifying no particular pattern between the age groups and cranial indices.

## Conclusion

The study indicated that most Ghanaian adults belong to the mesocephalic category of CI. Females had a higher CI compared to their male counterparts, which could be used to differentiate gender groups. Particularly, the range of CI was observed to be 67.4–89.5 in males with a mean of 77.3 ± 3.6 and a range of 72.9–92.6 in females with a mean of 79.0 ± 3.3. This information can be useful for forensic medicine, plastic surgeries for clinical and research purpose.
